# Cardiopulmonary exercise testing in mild generalized myasthenia gravis: an exploratory pilot feasibility study

**DOI:** 10.3389/fphys.2026.1760707

**Published:** 2026-03-10

**Authors:** Frauke Stascheit, Maximilian Lauermann, Lucie Risch, Frank Mayer, Sarah Hoffmann, Andreas Meisel, Kai Roecker, Michael Cassel

**Affiliations:** 1 Charité — Universitätsmedizin Berlin, Department of Neurology with Experimental Neurology, Berlin, Germany; 2 Neuroscience Clinical Research Center, Charité — Universitätsmedizin Berlin, Berlin, Germany; 3 University Outpatient Clinic for Sports Medicine and Sports Orthopedics, University of Potsdam, Potsdam, Germany; 4 Center for Stroke Research Berlin, Charité — Universitätsmedizin Berlin, Berlin, Germany; 5 Institute for Applied Health Promotion and Exercise Medicine (IfAG), Furtwangen University, Furtwangen, Germany

**Keywords:** cardiopulmonary exercise testing, fatigue, myasthenia gravis, neuromuscular disease, physical activity

## Abstract

**Introduction:**

We conducted an exploratory pilot feasibility study to assess the safety and feasibility of cardiopulmonary exercise testing (CPET) in patients with mild generalized myasthenia gravis (gMG), aiming to determine whether test termination results from systemic exertion or MG-specific neuromuscular fatigability and to evaluate short-term effects on fatigue.

**Methods:**

Nine patients with mild gMG (median age of 58 years, IQR 46.0–66.5) were included in this exploratory feasibility study and underwent a single maximal incremental CPET on a cycle ergometer with breath-by-breath gas exchange analysis and blood lactate measurements. Predefined exertion criteria (EC) included respiratory exchange rate (RER; ≥1.1), ventilatory equivalent for O_2_ (EQO_2_; ≥30), attainment of age-related maximum heart rate (ARMHR), peak blood lactate concentration (>6 mmol·L^−1^), and Borg Scale for Rated Perceived Exertion (RPE; ≥17). Repetitive nerve stimulation (RNS) was performed before and after CPET to assess neuromuscular fatigability. Fatigue was assessed using the Chalder Fatigue Scale (CFS) at baseline and 4 weeks after CPET.

**Results:**

CPET was completed by all participants without adverse events. All reported symptoms resolved within 30 min after exercise termination, and no participant required medical intervention. All participants completed the maximal incremental CPET according to the predefined protocol. Among the predefined EC, 44% of participants achieved an RER ≥1.1, 78% an EQO_2_ ≥30, 44% an ARMHR, 67% reached a peak blood lactate concentration >6 mmol·L^−1-^, and 67% an RPE ≥17. A decremental response in RNS was observed in 78% both before and after CPET. No relevant changes in CFS scores were detected over 4 weeks.

**Discussion:**

CPET appears safe and feasible in mild gMG, with exercise termination primarily driven by systemic exertion rather than MG-specific neuromuscular fatigue. CPET may complement clinical evaluation by supporting individualized training recommendations and the identification of cardiopulmonary limitations.

## 1 Introduction

Myasthenia Gravis (MG) is a rare, autoimmune disease of the neuromuscular junction leading to exercise-dependent muscle fatiguability of the ocular, bulbar, extremity and trunk, and muscles. Beyond skeletal muscle fatigability, MG may also involve respiratory muscle weakness and disturbances of autonomic or cardiovascular regulation (Peric et al., 2011; Zawadka-Kunikowska et al., 2023; Gilhus et al., 2019). Respiratory involvement may manifest as impaired ventilatory capacity or altered breathing patterns, while cardiovascular autonomic dysfunction has been reported in MG and may contribute to exercise intolerance and symptom perception during physical exertion. Pathogenic autoantibodies (Abs) to acetylcholine receptors (AChR), muscle-specific tyrosine kinase (MuSK), and lipoprotein-related proteins 4 (LRP4) are directly pathogenic at the neuromuscular endplate, although a subgroup of patients remains seronegative (Gilhus et al., 2019). Despite substantial advances in symptomatic and immunomodulatory treatments (Wiendl et al., 2024), up to 15% of patients experience highly active or refractory disease courses, often with significant impact on mobility, fatigue, and quality of life (Hoffmann et al., 2016; Lehnerer et al., 2022). Furthermore, the lower energy expenditure increases the risk for chronic lifestyle related diseases and premature mortality by 20%–30% in long-term (Warburton and Bredin, 2017; Westerberg and Punga, 2020). At the same time, fatigue and fluctuating muscle weakness often limit participation in exercise, resulting in a vicious circle of inactivity (Misra et al., 2020). Physicians have historically been reluctant to recommend structured exercise for MG patients due to concerns of overstraining already weak muscles, even though controlled evidence for exercise-related harm is lacking (Anziska and Sternberg, 2013).

Emerging data from small studies suggest that resistance and aerobic training in patients with mild generalized MG (gMG) can be performed safely and may improve fatigue, mobility, mood, and overall quality of life (Birnbaum et al., 2021; Gilhus, 2021; Oorschot et al., 2025; Voorn et al., 2025). However, despite increasing interest in exercise interventions, no evidence-based exercise recommendations or consensus guidelines currently exist for MG, and the role of objective exercise testing in this population remains insufficiently studied. In this context, assessment of physical performance in MG has largely relied on clinical scores and functional tests (de Meel et al., 2019; Bedlack et al., 2005), whereas objective physiological exercise testing has rarely been applied.

Cardiopulmonary exercise testing (CPET) enables an integrated assessment of the cardiovascular, pulmonary, and muscular systems during exercise and is increasingly used in neuromuscular diseases (NMD). A recent systematic review demonstrated that CPET is feasible in ambulatory patients with NMD and provides valuable insight into exercise limitation; however, the authors highlighted substantial heterogeneity in protocols, outcome measures, and criteria used to define maximal effort, underscoring the need for disease-adapted standards (Barroso de Queiroz Davoli et al., 2021).

More recent work has expanded the application of CPET-derived parameters in NMD, particularly in individuals with limited mobility, highlighting that CPET can provide additional pathophysiological insights beyond peak exercise responses (Blumberg et al., 2025). In parallel, a recent ENMC consensus workshop emphasized the role of objective outcome measures, including CPET-derived parameters, for exercise studies in muscle diseases, while identifying ongoing gaps regarding standardized outcome selection and interpretation across different neuromuscular conditions (Voorn et al., 2025). Despite these advances in NMD research, disease-specific data on the feasibility, safety, and interpretation of maximal incremental CPET in MG remain scarce.

The potential value of CPET in MG patients lies in improving the physiological understanding of exercise intolerance by differentiating between systemic cardiorespiratory limitation and disease-specific neuromuscular fatigability. Beyond this, CPet allows objective quantification of functional exercise capacity and may support individualized dosing of physical activity by defining safe and effective training intensities, while helping to identify non-neuromuscular contributors to reduced exercise capacity. However, it remains unclear whether exercise termination during CPET in MG primarily reflects systemic cardiorespiratory limitation or MG-specific fatigability.

We therefore conducted an exploratory pilot feasibility study with descriptive analyses to evaluate the safety, feasibility, and physiological characteristics of maximal incremental CPET in patients with mild gMG. Our primary aim was to determine whether termination of maximal incremental CPET in MG is driven by systemic exertion rather than MG-specific neuromuscular fatigability. As secondary aim, we evaluated the mid-term (4-week) effects of CPET on physical and mental fatigue with the *a priori* hypothesis that CPET would not worsen fatigue. By providing initial feasibility and safety data, this study may inform future recommendations for clinical testing and individualized exercise recommendations in MG.

## 2 Materials and methods

### 2.1 Standard protocol approvals, registrations, and patient consents

The study was approved by the ethics committee of the Charité–Universitätsmedizin Berlin (EA4/205/21). All participants provided written informed consent in accordance with the Declaration of Helsinki in its currently applicable form. The study followed the Strengthening the Reporting of Observational Studies in Epidemiology (STROBE) reporting guidelines.

### 2.2 Patients and study design

Patients with AChR-Abs positive mild gMG without bulbar or respiratory affection according to the Myasthenia Gravis Foundation of America (MGFA) clinical classification (MGFA IIA) were prospectively recruited at the Departments of Neurology at the Charité- Universitätsmedizin Berlin, which is a certified German “Integrated MG Centers of Excellence” (iMZ), accredited by the German Myasthenia gravis Society. CPET took place at the University Sports Medicine Outpatient Clinic in the University of Potsdam, Germany. Diagnosis of MG was based on international guidelines (Narayanaswami et al., 2021; Wiendl et al., 2024). Consecutively, patients were screened at the iMZ clinic from March 2022 to October 2022, independent of disease duration (Jaretzki et al., 2000). Exclusion criteria were (1) patients with gMG <18 years or >75 years of age; (2) patients with moderate or severe MG (MGFA >II); (3) patients with predominantly oropharyngeal or respiratory symptoms (MGFA IIB); (4) absolute contraindications for CPET: Acute myocardial infarction, myocarditis, pericarditis, endocarditis, decompensated heart failure, severe aortic stenosis, unstable angina pectoris, severe hypertension with systolic blood pressure (SBP) ≥200 mmHg or diastolic blood pressure (DBP) ≥110 mmHg, pulmonary embolism and relative contraindications for CPET: subacute phase after myocardial infarction or stroke, left main coronary artery stenosis, insufficiently controlled arrhythmias, 2nd or 3rd degree atrioventricular block, (3) moderate dyspnea (Grade 2) according to the Medical Research Council-Scale, (3) other musculoskeletal or neurological comorbidities that affect performance during CPET until voluntary exhaustion (Hollmann et al., 2006). Psychological symptoms were assessed using the Hospital Anxiety and Depression Scale (HADS), a validated self-report questionnaire consisting of two subscales for anxiety and depression, each ranging from 0 to 21, with higher scores indicating greater symptom severity (Bjelland et al., 2002). The HADS was administered at baseline to screen for clinically relevant anxiety or depressive symptoms.

### 2.3 Clinical assessment

Age, sex, disease duration, BMI, history of myasthenic crisis, current MG-specific medication (cholinesterase inhibitors, glucocorticoids, and long-term immunosuppressants), history of thymectomy, and comorbidities. Disease severity was assessed using the current MGFA classification and the quantitative MG (QMG)-score. Repetitive nerve stimulation (RNS) was performed of N. facialis (NF, M. orbicularis oculi) and N. accessorius (NA, M. trapezius pars descendens) to detect the MG-characteristic decrement before and within 10 min after CPET termination to evaluate exercise induced (local) muscle fatigability. Participants were asked not to take symptomatic therapy with pyridostigmine >4 h before the measurement in order to detect decremental response.

The Chalder Fatigue Scale (CFS) was used to assess the impact of CPET on fatigue (Cella and Chalder, 2010). It captures perceived severity of physical (items 1–7) and mental fatigue (items 8–11) over the past 4 weeks. The Likert total score ranges 0–33 with higher scores indicating greater fatigue. The bimodal (‘caseness’) score is derived by recoding responses (0–1 = 0; 2–3 = 1) yielding a 0–11 total, scores ≥4 indicate clinically relevant fatigue (Allen and Seaman, 2007). Assessment of CFS took place before CPET and 4 weeks after CPET.

### 2.4 CPET protocol

All participants performed a one-time stepwise incremental exercise test until voluntary exertion on a stationary bicycle ergometer (Excalibur Sport, Lode B.V., The Netherlands), lactate concentration measurements in capillary blood samples from the hyperaemized earlobe (Biosen, Magdeburg, Germany), and breath gas analysis (METALYZER 3B, CORTEX Biophysik GmbH, Germany).

The test protocol was conducted to established recommendations in sports medicine ((Roecker et al., 2003; Faude et al., 2009; Glaab and Taube, 2022). Following a 3-min familiarization and warm-up cycling (8 Watts, W), all participants started at 25W with a cadence at 70–80 revolutions per minute and with step increments of 25W every 3 min until exhaustion. Blood lactate measurements were collected as a complementary physiological parameter supporting high metabolic stress at test termination, thereby further strengthening the interpretation of maximal exertion in gMG. Step increment duration of 3 min was chosen allowing for the reliable measurement of lactate kinetics (Roecker et al., 2003; Faude et al., 2009). Blood samples (obtained from the earlobe), heart rate (ECG), and blood pressure (manual measurement with blood pressure cuff) were measured at rest, at the end of every 3-min stage, immediately at test termination and at 3- and 5-min post exercise. Additionally, ratings of perceived exertion (RPE) were assessed using the Borg 6–20 scale by finger-pointing on a printed scale in front of the participant at every measurement timepoint regarding (1) breathing, (2) muscles of the lower limbs, and (3) overall subjective exertion (Hollmann et al., 2006).

Reasons for individual termination of CPET (e.g., systemic exertion or dyspnea) were determined based on the participant’s self-reported primary limiting symptom at the time of exercise cessation and were documented immediately after test termination using standardized questioning by the supervising investigator. This information was complemented by the investigator’s clinical assessment based on CPET-derived parameters. By protocol, CPET had to be terminated in one of the following scenarios: ≥10 mmHG drop in SBP in combination with other signs of myocardial ischemia, SBP ≥280 mmHG, dizziness, pre-syncope, or more severe neurological signs, cyanosis, paleness, participant wants to terminate or is not able to keep 70 rpm (Glaab and Taube, 2022). To ensure safety during the CPET, the test was conducted by qualified medical staff and a sports physician was available at all times.

### 2.5 Outcome measures

Feasibility of maximal incremental CPET was assessed using predefined domains rather than a single outcome measure. These domains included (1) safety, (2) protocol completion and adherence, and (3) physiological test quality. Independent of feasibility, secondary outcome measures were used to explore the potential influence of CPET on physical and mental fatigue over a 4-week follow-up period. Safety was evaluated based on the occurrence of adverse events, predefined termination criteria, and the need for medical intervention during or after CPET. Protocol completion and adherence were assessed by the proportion of participants who completed the CPET according to the predefined protocol without premature termination. Physiological test quality was evaluated by descriptive assessment of CPET parameters and attainment of neuromuscular disease–specific criteria for maximal cardiorespiratory effort.

### 2.6 Attainment of exertion criteria

Breath-by-breath data were recorded continuously throughout exercise and recovery. Presented values were taken as the reported value out of the 9-panel graphical array (Glaab and Taube, 2022). Peak oxygen uptake (VO_2_peak) was defined as the highest oxygen uptake achieved during exercise. The ventilatory equivalent for oxygen (VE/VO_2_) at the time of VO_2_peak was reported separately and used as a criterion to support attainment of maximal effort. Peak heart rate was defined as the highest heart rate recorded during the test. Peak respiratory exchange ratio (RERpeak) was defined as the highest value achieved during exercise. Ratings of perceived exertion were recorded at peak exercise using the Borg 6–20 scale.

Maximal cardiorespiratory effort during CPET was assessed using a combination of physiological and perceptual criteria. To distinguish if people with mild gMG terminate the CPET due to probable MG-specific muscle fatiguability or reach endpoints that reflect maximal exertion in general, we defined the following exertion criteria: (1) respiratory exchange rate (RER) ≥1.1, (2) ventilatory equivalent of oxygen (EQO2) ≥30, (3) ≥100% of age-related maximum heart rate (ARMHR, 208–0.7 x age in years), (4) maximum blood lactate concentration (≥6 mmol·L^-1^) and (5) Ratings of general perceived exertion (RPE ≥17) being a self-reported measurement tool to evaluate PA intensity. The scaling ranges from “6” (“no exertion at all”) to “20” (“maximal exertion”), with “17” (“very hard”) considered as exertional criterion (Hollmann et al., 2006; Borg, 1982; Howley et al., 1995; Tanaka et al., 2001; Glaab and Taube, 2022).

All available CPET parameters were evaluated descriptively with respect to these criteria. Given the exploratory pilot feasibility design and the small sample size, attainment of maximal effort was not used as an inclusion criterion but served to support the interpretation of CPET termination and overall test quality.

### 2.7 Statistics

Given the exploratory pilot feasibility design, all CPET-derived variables were analyzed descriptively. Peak values for the EQO2 at VO_2_ peak, heart rate, respiratory exchange ratio, and perceived exertion were summarized using medians and interquartile ranges. Attainment of criteria for maximal effort was summarized using counts and proportions. Feasibility was not determined by a single parameter but was evaluated based on the overall pattern across predefined domains, including safety, protocol completion, and physiological test quality. No inferential statistical testing was performed. Continuous data are presented as mean and standard deviation as well or median and interquartile range (IQR) where appropriate, categorical variables as absolute frequencies and percentages. Data acquisition and data analysis of CPET was carried out by MetaSoft Studio (Version 5.12.0, custo med GmbH, Germany) and custo diagnostic (Version5.4.6, custo med GmbH, Germany). All statistical analyses were performed in Prism (version 10.4, GraphPad Software, San Diego, CA, USA). Graphics of the CPET were extracted from the automatically generated Wassermann’s 9-panel plot MetaSoft Studio (Version 5.12.0) and custo diagnostic (Version 5.4.6).

## 3 Results

### 3.1 Participant characteristics

A total of nine patients with mild gMG were included in this exploratory pilot feasibility study. Baseline clinical and demographical characteristics are summarized in Table 1. In summary, median age was 58 (IQR 46.0–66.5) years, 44% (n = 4) were of female sex and median disease duration was 12 (IQR 0.5–61.0) months. Median BMI was 26.7 (IQR 22.6–29.2). Five patients were AChR-Abs positive (56%) and four patients (44%) were seronegative as defined by negative testing for AChR, MuSK- and LRP4 Abs. None were double-positive for Abs against MuSK or LRP4. Disease severity as measured by MG-ADL score with a median of 4 points (IQR 1.5–6.0). Previous thymectomy was performed in the majority of patients (78%, n = 7), 56% (n = 5) were treated with immunosuppressive drugs and/or low dose steroids. No patient had a history of myasthenic crisis. Mild fatigue was observed at baseline (median CFS 13, IQR 9.0–21.5), with no clinically relevant depressive symptoms on HADS.

**TABLE 1:** Clinical and demographical characteristics.

Number of patients	9
Age (years), *median (IQR)*	58 (46.0–66.5)
Early-onset myasthenia gravis, *n (%)*	6 (67%)
Female sex, *n (%)*	4 (44%)
Disease duration (months), *median (IQR)*	12 (0.5–61.0)
Weight (kg), *median (IQR)*	76.8 (70.0–87.5)
BMI, *median (IQR)*	26.7 (22.6–29.2)
Antibody status, *n (%)* AChR-Abs+ Seronegative	5 (56%) 4 (44%)
Previous thymectomy, *n (%)*	7 (78%)
Thymoma, *n (%)*	1 (11%)
Thymic hyperplasia	3 (33%)
History of myasthenic crisis, *n (%)*	0 (0%)
Total MG-ADL score, *median (IQR)*	4 (1.5–6.0)
Total MG-QoL15r score, *median (IQR)*	17 (5.5–24.0)
Total chalder fatigue score, *median (IQR)*	13 (9.0–21.5)
Total HADS score, *median (IQR)*	3.5 (1.0–6.0)
Myasthenia gravis therapy at baseline, *n (%)*	
Pyridostigmine	9 (100%)
Any steroid	2 (22%)
Standard immunosuppressive therapy^a^	3 (33%)
Intensified therapy^b^	0 (0%)
Steroid and any immunosuppressive therapy	0 (0%)
No steroid and no immunosuppressive therapy	3 (33%)

Abbreviations*:* AChR-Abs, acetylcholine-receptor antibody; HADS, Hospital Anxiety and Depression Scale; MG-ADL, myasthenia gravis activity of daily life score; MG-QoL15r, myasthenia gravis quality of life score; N, number of participants.

^a^ Standard immunosuppressions include azathioprine, mycophenolate mofetil, methotrexate.

^b^ Intensified therapy include rituximab, complement-inhibitors, FcRn-inhibitors.

### 3.2 Feasibility of maximal incremental CPET

#### 3.2.1 Safety

No adverse events occurred during or after CPET. All reported symptoms resolved within 30 min after exercise termination, and no participant required medical intervention.

#### 3.2.2 Protocol completion and reasons for termination

All participants completed the maximal incremental CPET according to the predefined protocol. The most frequently reported reason for test termination was subjective exertion of the lower limb muscles (67%, n = 6), followed by dyspnea (22%, n = 2). In one participant, dizziness led to test termination at a workload of 100 W. Reasons for termination were based on participant-reported limiting symptoms and were corroborated by the supervising investigator.

### 3.3 Physiological test quality and attainment of exertion criteria

The results of the CPET with a mean maximum power output of 144 W (SD 37 W), mean relative power output of 1.6 W·kg-1 (SD 0.3 W·kg-1) and mean absolute V̇O2peak of 2.0 L·min-1 (SD 0.4 L·min-1) are presented in Table 2. The predefined exertion criteria reached varied in the cohort from 1 to 5 out of the five endpoints (n = 2/1/2/2/2 for achieved endpoint sum of 1/2/3/4/5 criteria, Table 3). In 44% (n = 4) RER ≥1.1, in 78% (n = 7) EQO_2_ ≥30, in 44% (n = 4) ARMHR, in 67% (n = 4) peak blood lactate concentration >6 mmol·L^-1-^, and in 67% (n = 6) RPE ≥17 was reached by the patients, indicating that the EQO_2_ criteria was the most often achieved endpoint (Table 3). A decremental response in RNS was observed in 78% both before and after CPET. No relevant changes in CFS scores were detected over 4 weeks.

**TABLE 2:** Result of cardiopulmonary exercise testing.

Subject	1	2	3	4	5	6	7	8	9
BP at rest (mmHg)	135/80	115/75	130/80	135/80	115/80	135/90	150/80	130/70	120/80
BP max (mmHg)	170/90	165/90	180/90	180/90	170/90	185/100	190/80	160/70	160/100
HR at rest (bpm)	60	57	79	68	75	65	57	77	58
HR max (bpm)	145	161	166	150	190	171	190	155	170
P max (W)	125	175	125	125	150	100	225	125	150
P max rel. (W/kg body weight)	2.08	2.28	1.26	1.15	2.00	0.50	2.65	1.36	1.97
P at IAT (W)	80	117	83	83	83	59	152	91	83
P at IAT rel. (W/kg body weight)	1.3	1.5	0.8	0.9	1.1	0.8	1.8	1.2	1.1
EQO_2_ at V̇O_2_ peak	39	33	33	28	34	32	31	29	33
EQCO_2_ at V̇O_2_ peak	37	34	36	30	33	31	31	30	33
V̇O_2_max calculated (L/min)	1.92	2.56	2.06	1.78	2.27	1.89	3.17	1.78	2.27
V̇O_2_ peak measured (L/min)	1.86	2.45	1.89	1.91	2.12	1.43	3.08	1.77	2.19

Abbreviations: N, number of participants; BP, Blood Pressure; HR, heart rate; max, maximal; EQO_2_, ECO_2_, ventilatory equivalents for O_2_ and CO_2_ respectively; IAT, Individual Anaerobic Lactate Threshold (“LT2”).

**TABLE 3:** Attainment of exertion criteria.

Subject [N]	1	2	3	4	5	6	7	8	9
RER	1.10	1.04	0.96	0.99	1.21	1.10	1.14	1.08	1.09
EQO_2_ at V̇O_2_ peak	39	33	33	28	34	32	31	29	33
ARMHR/HR max (bpm)	167/145	175/161	164/166	158/150	186/190	171/171	165/190	159/155	177/170
Max. Blood lactate (mmol·L^-1^)	6.3	5.9	5.4	6.9	9.8	4.8	6.9	6.9	10.3
RPE	16	14	19	15	19	18	18	17	19
Endpoints reached [x/5]	3	1	4	1	5	4	5	2	3

Abbreviations: AChR-Abs, acetylcholine-receptor antibody; HADS, Hospital Anxiety and Depression Scale; MG-ADL, myasthenia gravis activity of daily life score; MG-QoL15r, myasthenia gravis quality of life score; N, number of participants.

Abbreviations: ARMHR, age-related maximum heart rate; HR, heart rate; RPE, Ratings of perceived exertion; EQO2, ventilatory equivalent for O2. Grey boxes indicate reached predefined exertion criteria.

**FIGURE 1:**
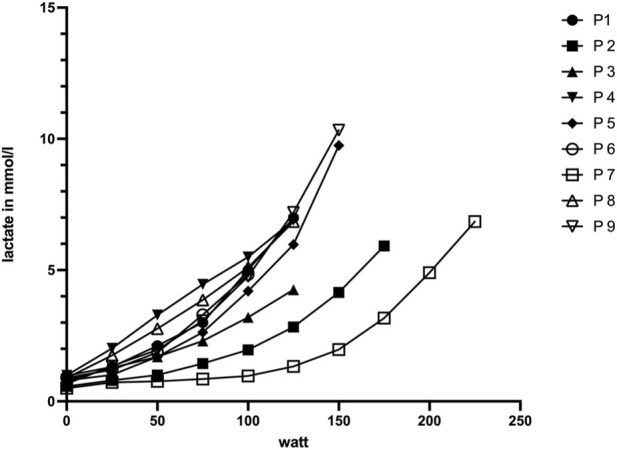
Blood lactate concentration in relation to the power output during bicycle ergometry. Individual blood lactate levels (mmol·L^−1^) are plotted against the corresponding power output (W) during incremental CPET on a bicycle ergometer. The figure illustrates the increase of lactate concentrations with rising workload, reflecting normal physiological response.

In addition, peak blood lactate concentrations indicated high metabolic stress at test termination, further supporting the interpretation that exercise cessation occurred under near-maximal physiological conditions. Furthermore, Figure 1 shows typical patterns of load-dependant lactate concentrations during increasing increments, potentially allowing for determination of training recommendations. An overview of individual CPET parameters and criteria fulfillment is provided in Tables 2, 3.

### 3.4 Decremental response persists despite CPET in mild gMG

Before CPET, we could detect decremental response in both, M. orbicularis oculi and M. trapezius in 78% (n = 7) MG patients, which was also detectable immediately after CPET in the same patients. All of these patients were seropositive for AChR-Abs.

### 3.5 Fatigue did not increase over time

**FIGURE 2:**
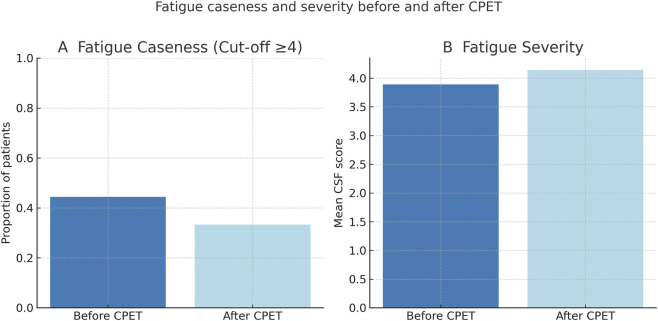
Fatigue caseness and severity before and after CPET. Panel **(A)** shows the proportion of MG patients classified as fatigued (caseness), defined by a Chalder Fatigue Scale (CSF) cut-off of ≥4, before and after CPET. Panel **(B)** displays mean CSF fatigue severity scores at the same time points. No clinically relevant worsening of fatigue was observed immediately after CPET.

**FIGURE 3:**
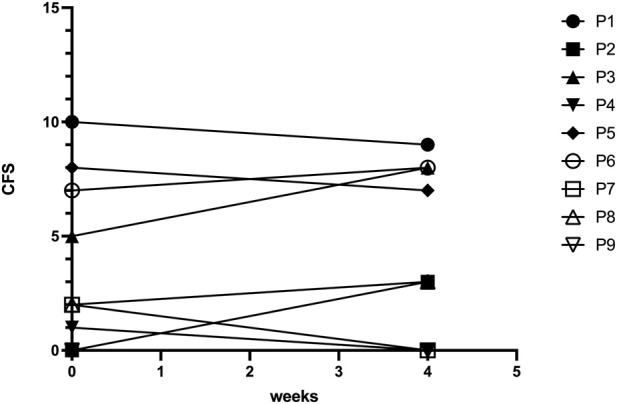
Individual fatigue scores before and after CPET. Spaghetti plots illustrate individual Chalder Fatigue Scale (CSF) scores for each patient before and 4 weeks after CPET. While some variability between patients was observed, there was no consistent increase in fatigue severity across the cohort.

Secondary outcome analyses were exploratory. All nine participants completed the CFS prior to CPET (M1). Four individuals (44%) reported clinically significant fatigue, defined as a CFS score ≥4. At M2, after 4 weeks, no relevant changes in fatigue severity or caseness were observed (Figure 2). Figure 3 illustrates individual trajectories, showing variability between patients but no consistent increase in fatigue severity across the cohort. Moreover, MG-ADL scores as a measure of disease severity did not change significantly before and after CPET (median baseline MG-ADL score: 4.0 [IQR 1.5–6.0]; median post-CPET score: 3.0 [IQR 1.0–7.0]).

## 4 Discussion

In this exploratory pilot feasibility study, we demonstrate that cardiopulmonary exercise testing on a bicycle ergometer is safe and feasible in patients with mild generalized MG. Exercise limitation was consistently determined by systemic exertion rather than MG-specific muscle fatigability, and no worsening of fatigue symptoms was observed during follow-up. The concordance between cardiorespiratory effort criteria and elevated lactate levels further strengthens the case for using stepwise increased CPET protocols as a feasible and informative tool to assess exercise capacity in patients with mild gMG. Beyond supporting near-maximal physiological stress at test termination, the observed load-dependent lactate kinetics demonstrate that lactate-based threshold determination (individual anaerobic threshold) is feasible in patients with mild gMG. This suggests that CPET may not only serve as an objective assessment tool but could also allow the derivation of individualized training recommendations, analogous to standardized sports medical evaluations in healthy or exercise-naïve populations. Importantly, feasibility was not defined by a single parameter but by a consistent pattern across safety, protocol completion, and physiological test quality, supporting the applicability of CPET in patients with mild gMG. These findings indicate that individualized training intensities can be derived from CPET results without imposing additional disease-specific health risks.

While advances in biological therapies have substantially improved quality of life in MG (Howard et al., 2021b; Howard et al., 2021a; Vu et al., 2022), lifestyle-related factors such as physical activity remain important contributors to long-term health and functional capacity. In contrast to other autoimmune disorders, including multiple sclerosis and rheumatoid arthritis, where structured exercise is an established adjunct therapy (Sharif et al., 2018), evidence-based recommendations for physical training in MG remain limited. Available studies suggest that aerobic and resistance exercise can be performed safely in patients with stable MG and may improve functional outcomes such as muscle strength, mobility, and fatigue perception (Birnbaum et al., 2021; Gilhus, 2021). However, most of these studies relied on submaximal exercise protocols, functional tests, or patient-reported outcomes, while objective physiological assessment of maximal exercise capacity has rarely been incorporated. In neuromuscular diseases more broadly, cardiopulmonary exercise testing has been shown to be feasible and informative when interpreted using disease-adapted criteria, allowing differentiation between systemic cardiorespiratory limitation and neuromuscular contributors to exercise intolerance (Barroso de Queiroz Davoli et al., 2021; Veneman et al., 2024; Voorn et al., 2025). Against this background, our findings extend the existing literature by demonstrating that patients with mild gMG are able to undergo maximally incremental CPET safely and achieve expected maximal physiological responses. The concordance between cardiorespiratory effort criteria achieved and elevated blood lactate concentrations supports the interpretation that exercise termination was driven by systemic exertion rather than premature cessation due to focal neuromuscular fatigability. This supports the use of CPET not only as a research tool but also as a structured framework for individualized exercise prescription in MG, moving beyond empirically derived recommendations—such as avoiding prolonged endurance exercise or training only under specific environmental conditions—which are currently not supported by robust physiological evidence (Cass, 2013). Moreover, patients with mild gMG were able to perform a stepwise incremental lactate testing protocol with typical load-dependent lactate kinetics, indicating that lactate-based threshold determination may be feasible and could inform individualized training recommendations under supervised conditions.

Our findings extend prior work by incorporating maximum incremental CPET, to provide objective cardiopulmonary measures that complement patient-reported outcomes and build on studies using submaximal protocols. In our cohort of nine participants, we found great heterogeneity in the attainment of predefined exertion criteria. Participants met one (22%), two (11%), three (33%), four (11%), and five (22%) out of five possible exertion criteria. These findings indicate substantial interindividual variability in exercise tolerance within the mild gMG population. Six out of nine participants (67%) met ≥3 exertion criteria.

Furthermore, the same heterogeneity shows up in the comparison of CPET results to normal standard values (Tomlinson et al., 2022; Scharhag-Rosenberger et al., 2009). 56% of the cohort met the standard values for healthy individuals regarding relative power and V̇O2max. These participants also reached ≥3 exertion criteria. In conjunction with the 78% positive test results of the RNS, which ensure the existence of neuromuscular transmission disorder, it is notable how long the MG patients could comply to the requirements of the CPET. Our protocol incorporated stages of 3 min and increments of 25W and therefore took in mean approximately 18 min (6 stages x 3 min) to reach an absolute power output of 144W. Test termination was predominantly driven by systemic cardiopulmonary limitation rather than MG-specific neuromuscular fatigability, supported by unchanged RNS findings before and after CPET. The duration of the CPET represents an additional methodological consideration. Due to the use of a stepwise protocol with 25 W increments and a step duration of 3 min, total exercise duration was longer than the commonly recommended 8–12 min for CPET ramp protocols and averaged with approximately 18 min. This enabled for the valid measurement of blood lactate concentrations and the determination of the individualized anaerobic threshold (IAT) during exercise in parallel (Roecker et al., 2003; Faude et al., 2009). Before the start of the study, the performance profiles of the subjects were unknown and a load scheme with the greatest possible controllability, interpretability and safety was selected. It is generally intended to end up with approximately six increments until exhaustion to allow for calculating the IAT values. As a result, the test subjects exceeded the previously assumed load limit in a positive sense. Even if the maximum values may have been lower than possible due to the longer load duration, the load criteria shown in Table 2 can be regarded as even more meaningful in this context.

This study adds to the growing body of evidence that people with MG can adhere safely to endurance exercise (O'Connor et al., 2020; Karaahmet et al., 2022). As people with gMG avoid high demanding physical activity, fearing clinical deterioration of myasthenic syndromes and worsening of fatigue (Karaahmet et al., 2022) safe clinical settings like CPET can be used to investigate cardiopulmonary fitness and to give individualized training recommendations for basic endurance training relative to submaximal reference points as the anaerobic thresholds from breath gas analysis or simply from blood lactate measurements. Although there are only a few studies with a primary focus on exercise intervention in MG, all of them showed that exercise was feasible for most patients with mild gMG, regardless if aerobic training or progressive resistance training was performed.(Rahbek et al., 2017; Punga et al., 2023; Westerberg et al., 2017). One study even examined if disease specific miRNA may be influenced by physical activity and found a reduction in disease-specific microRNAs miR-150-5p and miR-21-5p after the training period (Westerberg et al., 2017).

Thus, implementation of CPET to provide patients with individualized training recommendations may improve cardiopulmonary fitness as well as quality of life and reduce common cardiovascular risk factors. Moreover, patients with mild gMG can undergo maximum incremental CPET for suspected cardiopulmonary diseases without MG-specific restrictions. However, these findings are limited to patients with mild gMG (MGFA class II) and cannot be generalized to patients with more advanced disease stages.

### 4.1 Limitations

This study has several limitations that should be considered when interpreting the results. First, the exploratory pilot design and the small sample size give the study a case series–like character and limit the generalizability of the findings. Only patients with mild gMG were included, and conclusions cannot be extended to patients with more severe disease, different clinical phenotypes, or unstable disease courses. In addition, the single-center design and the absence of a control group preclude comparisons with healthy individuals or other neuromuscular populations.

A further limitation relates to the comparability of the protocol used for assessment of maximal cardiorespiratory effort. According to the more recently proposed NMD–specific criteria for maximal cardiorespiratory effort by Veneman et al. (Veneman et al., 2024), 6 of 9 participants in the present study would have fulfilled at least two of the secondary criteria (peak RER ≥1.10, peak heart rate ≥85% of predicted maximum, and/or peak rating of perceived exertion ≥17). This supports the attainment of maximal effort in most of the cohort analysed and suggests similar achievements by use of a stepwise increased CPET protocol including measures of lactate kinetics. However, the comparability of the outcome parameters to the disease-adapted criteria is limited due to significant differences in the CPET protocols.

Methodological limitations should also be acknowledged. Reasons for test termination were partly based on participant-reported limiting symptoms, which may introduce subjective bias, although these reports were corroborated by physiological parameters and investigator assessment. Moreover, most participants were not accustomed to high-intensity endurance exercise, which may have influenced perceived exertion, particularly during the early stages of the test. However, lactate concentrations measured support the feasibility the methodological approach in this cohort. Finally, only a single CPET session was performed, preventing assessment of intra-individual variability or test–retest reliability. Taken together, these limitations underscore that the present study was designed to assess feasibility, safety, protocol adherence, and physiological response patterns rather than to provide inferential or prognostic conclusions.

## 5 Conclusion

In this exploratory pilot feasibility study, CPET on a bicycle ergometer represents a safe and feasible modality to assess physiological exercise responses in patients with mild gMG, without disease-specific safety concerns in this cohort. Test termination was due to cardiopulmonary exertion rather than MG-specific muscle fatiguability. An acute bout of maximal physical activity did not lead to a worsening of fatigue symptoms 4 weeks following the exercise tests. Specific training intensity recommendations can therefore be derived from individual exercise test results without any additional acute or chronic MG specific health risks. Additionally, maximum incremental CPET may be recommended in patients suffering from mild gMG to test and diagnose for suspected cardiopulmonary diseases. Together, these results emphasize that CPET can complement clinical evaluation by enabling both individualized training recommendations and early detection of cardiopulmonary comorbidities. However, future intervention studies should be conducted to evaluate if training effects in patients with mild gMG based on CPET are comparable to the general population as well as to determine its effect on fatigue symptoms.

## Data Availability

The raw data supporting the conclusions of this article will be made available by the authors, without undue reservation.
